# Visit-to-visit HbA1c variability is associated with aortic stiffness progression in participants with type 2 diabetes

**DOI:** 10.1186/s12933-023-01884-7

**Published:** 2023-07-06

**Authors:** Qianhua Fang, Juan Shi, Jia Zhang, Ying Peng, Cong Liu, Xing Wei, Zhuomeng Hu, Lin Sun, Jie Hong, Weiqiong Gu, Weiqing Wang, Yifei Zhang

**Affiliations:** 1 Department of Endocrine and Metabolic Diseases, Shanghai Institute of Endocrine and Metabolic Diseases, Ruijin Hospital, Shanghai Jiao Tong University School of Medicine, Shanghai, China; 2 Shanghai National Clinical Research Center for Metabolic Diseases, Key Laboratory for Endocrine and Metabolic Diseases of the National Health Commission of the PR China, Shanghai Key Laboratory for Endocrine Tumor, State Key Laboratory of Medical Genomics, Ruijin Hospital, Shanghai Jiao Tong University School of Medicine, Shanghai, China

**Keywords:** Trajectory, HbA1c variability, FBG variability, Type 2 diabetes, Pulse wave velocity, Aortic stiffness

## Abstract

**Background:**

Glycemic variability plays an important role in the development of cardiovascular disease (CVD). This study aims to determine whether long-term visit-to-visit glycemic variability is associated with aortic stiffness progression in participants with type 2 diabetes (T2D).

**Methods:**

Prospective data were obtained from 2115 T2D participants in the National Metabolic Management Center (MMC) from June 2017 to December 2022. Two brachial-ankle pulse wave velocity (ba-PWV) measurements were performed to assess aortic stiffness over a mean follow-up period of 2.6 years. A multivariate latent class growth mixed model was applied to identify trajectories of blood glucose. Logistic regression models were used to determine the odds ratio (OR) for aortic stiffness associated with glycemic variability evaluated by the coefficient of variation (CV), variability independent of the mean (VIM), average real variability (ARV), and successive variation (SV) of blood glucose.

**Results:**

Four distinct trajectories of glycated hemoglobin (HbA1c) or fasting blood glucose (FBG) were identified. In the U-shape class of HbA1c and FBG, the adjusted ORs were 2.17 and 1.21 for having increased/persistently high ba-PWV, respectively. Additionally, HbA1c variability (CV, VIM, SV) was significantly associated with aortic stiffness progression, with ORs ranging from 1.20 to 1.24. Cross-tabulation analysis indicated that the third tertile of the HbA1c mean and VIM conferred a 78% (95% confidence interval [CI] 1.23–2.58) higher odds of aortic stiffness progression. Sensitivity analysis demonstrated that the SD of HbA1c and the highest HbA1c variability score (HVS) were significantly associated with the adverse outcomes independent of the mean of HbA1c during the follow-up.

**Conclusions:**

Long-term visit-to-visit HbA1c variability was independently associated with aortic stiffness progression, suggesting that HbA1c variability was a strong predictor of subclinical atherosclerosis in T2D participants.

**Supplementary Information:**

The online version contains supplementary material available at 10.1186/s12933-023-01884-7.

## Background

Cardiovascular disease (CVD) is well established as the primary cause of morbidity and mortality in individuals with diabetes. Previous observational studies have consistently reported a causal relationship between chronic hyperglycemia and adverse vascular outcomes in individuals with type 2 diabetes (T2D) [1–5]. Thus, the strategies for glycemia management in individuals with diabetes attempt to reduce blood glucose to target levels as soon as possible. However, intensive glycemic control failed to show beneficial effects on minimizing the risk of CVD events in T2D patients [6–8]. Furthermore, according to the Action to Control Cardiovascular Risk in Diabetes (ACCORD) trial, all-cause mortality in the intensive-therapy group was significantly higher than that in the standard-therapy group [9]. Therefore, in addition to focusing on glycemic levels, greater glycemic variability (i.e., reduced glycemic control) has recently been reported as an important correlate of the development of vascular complications in diabetes [10–12].

Glycemic variability is reflected by visit-to-visit glycemic excursions, and is divided into two components: short-term (days to weeks) and long-term (months to years) glycemic variability, which is measured by the fluctuation of glycated hemoglobin (HbA1c) or other metrics of glycemia [13]. A 5-year retrospective cohort study of 587 UK primary care practices demonstrated that glycemic variability was an important factor for mortality, suggesting that a stable glycemic level is associated with lower mortality risk, especially in older people with diabetes [14]. Moreover, an analysis of 240 consecutive T2D patients with well-controlled HbA1c showed that glycemic variability independently predicted the 10-year risk of CVD [15]. Nevertheless, these studies mainly focused on clinical CVD events, which usually affect people’s quality of life when these adverse outcomes occur. This observation raises the question of whether glycemic variability is a valid marker for the early stage of CVD in individuals with T2D.

Some studies have focused on the impact of glycemic variability on subclinical atherosclerosis, a precursor of clinical CVD. For example, a study reported that short-term glycemic variability assessed using continuous glucose monitoring (CGM) system data was associated with subclinical atherosclerosis in Chinese T2D patients [16]. However, other studies reported that no significant correlation was observed between aortic stiffness and short-term glycemic variability assessed using CGM in T2D patients [17, 18]. This apparent contradiction highlights the need to clarify the true association (or lack thereof) between glycemic variability and aortic stiffness. Additionally, in contrast to research on short-term glycemic variability, few studies have explored the effect of long-term glycemic variability on the risk of aortic stiffness progression in T2D participants. Therefore, the objective of this prospective study was to investigate the associations of long-term visit-to-visit variability in HbA1c or fasting blood glucose (FBG) with brachial-ankle pulse wave velocity (ba-PWV) changes, which is considered as the gold standard for assessing aortic stiffness in a simple and noninvasive way [19–21].

## Methods

### Participants and study design

This prospective study enrolled participants from the National Metabolic Management Center (MMC) in Ruijin Hospital, Shanghai Jiao Tong University School of Medicine, which is one of the diabetes care systems in China [22, 23]. Patients with T2D aged 18 years or older were recruited. A total of 5655 T2D patients were followed from June 2017 to December 2022. If possible, all participants underwent ba-PWV measurement by a trained independent observer at least three times within five years (i.e., baseline, year 2 or year 3, and year 5). The aortic stiffness status was determined according to the first and last ba-PWV levels for those with at least two ba-PWV measurements during the follow-up period in our study. Individuals with a follow-up period < 0.5 years (n = 750), missing baseline ba-PWV data (n = 416) or without second ba-PWV measurements (n = 2213), an ankle-brachial pressure index (ABI) < 0.9 (n = 44) [24], or less than 3 HbA1c or FBG measurements (n = 117) were excluded. A total of 2115 participants were finally included in the current study. Furthermore, after excluding participants with less than 5 HbA1c measurements (n = 721), 1394 participants were included in the calculation of the HbA1c variability score (HVS) as a part of the sensitivity analysis (Flow diagram seen in Supplemental Figure 1). Written informed consent was obtained from all participants, and the Institutional Review Board of Ruijin Hospital, Shanghai Jiao Tong University School of Medicine, approved the study protocol.

**Fig. 1:**
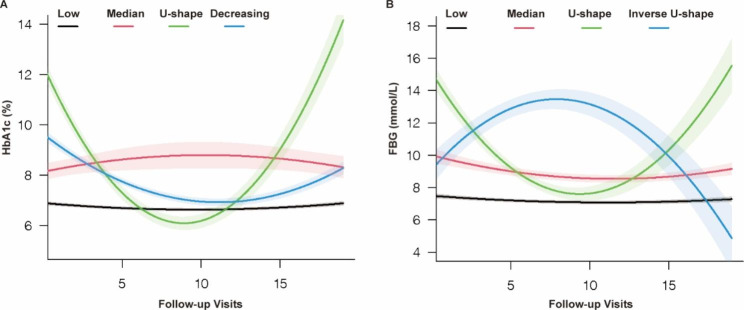
Trajectories of HbA1c and FBG during follow-up visits. Solid lines show class-specific mean predicted levels as a function of follow-up visits estimated from the best fitting model (4-class latent class growth mixed model), shading around the lines represent confidence bands for the calculated trajectory

### Clinical data collection

All participants completed a standardized questionnaire and underwent comprehensive clinical and laboratory examinations at registration and follow-up visits, with data recorded in an MMC-specialized electronic medical data record system, as previously described [25, 26]. Smoking status was defined as ‘ideal’ if the participants did not smoke or had quit smoking for more than 12 months. Drinking status was recorded as ‘yes’ for participants who drank weekly or almost weekly. Body mass index (BMI) was calculated as the ratio of body weight (in kilograms) to height (in meters squared) [27]. After at least 5 min of quiet rest, blood pressure (BP) and heart rate were measured using an automated electronic device. The urinary albumin/creatinine ratio (UACR) was calculated as the urinary albumin concentration divided by the urinary creatinine concentration. To assess fasting blood glucose (FBG), blood samples collected from participants after overnight fasting were measured. HbA1c was measured by high-performance liquid chromatography.

### Measures of glycemic variability

We recommended that all participants visit our center for two to four visits per year based on the MMC-related standard operation procedure; the actual frequency of follow-up visits was flexibly adjusted if necessary [28]. FBG and HbA1c were routinely measured at each visit. The follow-up recommendation provided us with multiple measurements of HbA1c and FBG over a relatively long period instead of in a short period. Using the three or more HbA1c and FBG measurements, the long-term visit-to-visit glycemic variability values were calculated, including the coefficient of variation (CV), successive variation (SV), variation independent of the mean (VIM), and average real variability (ARV) [29, 30]. The CV was calculated as the ratio of standard deviation (SD) to the mean; the SV was calculated as the square root of the average squared difference between successive measurements [31]; the VIM was calculated as the SD divided by the mean to the power x and multiplied by the population mean to the power x, with x derived from curve fitting [32]; the ARV was calculated as the average of the absolute differences between consecutive glycemia measurements [11] (details are provided in the Supplemental formula).

### Ba-PWV measurement

To obtain ba-PWV measurements, participants were allowed to rest for at least 5 min in a supine position, and then suitable cuffs were attached to their bilateral upper arms and ankles to obtain waveforms over the brachial and tibial arteries with an automated recording apparatus (BP-203RPE III, form PWV/ABI, Omron Healthcare Co.). The ba-PWV value was calculated as the transmission distance from the bilateral upper arms to the ankles divided by the transit time, as previously described [25]. We adopted the mean value of ba-PWV of the left and right sides for analysis.

In the study, participants were divided into two subgroups according to whether ba-PWV increased or persisted high and whether it reduced or persisted low on the last measurement during the whole follow-up period compared with the first measurement. To define this categorization, we divided the first and last ba-PWV measurements into quartiles and classified participants as having either increased their quartile distribution or persisted within the higher third and fourth quartile groups and those who decreased their quartile distribution or persisted within the lower first and second quartile groups (Supplemental Fig. 2) [33, 34].

### Statistical analysis

The multiple imputation of chained equations (MICEs) method was used to impute missing baseline covariate data for the aortic stiffness assessments during follow-up visits. Unsupervised cluster analysis using a multivariate latent class growth mixed model was applied to identify trajectories of HbA1c and FBG values over time [35]. We conducted a series of polynomial specifications of HbA1c and FBG as a function of follow-up visits with a class number ranging from 2 to 7. The optimal trajectories were determined based on the minimum Bayesian Information Criterion [36], while maintaining the posterior probabilities for all latent classes (> 0.70) and class size (≥ 2% of the population) [37]. We computed the odds ratio (OR) and 95% confidence interval (CI) for dichotomous subgroups of ba-PWV changes by assigned trajectory and glycemic variability using logistic regression models. Noteworthy, we adopted the natural logarithmic transformations for HbA1c and FBG variability metrics in logistic regression analysis to achieve more normal distribution. Three models that adjusted for major covariables were constructed: Model 1, which adjusted for age and gender; Model 2, which additionally adjusted for variables in Model 1 plus diabetes duration, systolic blood pressure (SBP), BMI, heart rate, ideal smoking, alcohol consumption, history of CVD, low-density lipoprotein cholesterol (LDL), triacylglycerols (TG), UACR, and the use of antihypertensive agents, lipid-lowering agents, insulin, or oral antidiabetic agents; and Model 3, which additionally adjusted for the average HbA1c during follow-up.

In addition, restricted cubic splines with three knots at the 10th, 50th, and 90th percentiles were used to flexibly model the association of glycemic variability with increased aortic stiffness and to describe the shape of the overall dose-response relationship [38]. The reference points in the analyses were the median values. Using splines, we estimated the multivariate-adjusted OR (95% CI) and created plots for a visual assessment of the relationships. Restricted cubic spline analyses were performed with Harrell’s regression modeling strategies (rms) package in R.

Sensitivity analyses were performed. We first assessed the association between aortic stiffness progression and the HVS, a new scale that reflects the frequency of HbA1c increases or decreases. The HVS was calculated as the number of changes in HbA1c more than 0.5% different than the value prior within an individual with at least five HbA1c measurements during the observational period [39]. Previous studies have demonstrated that HVS is associated with increased risks of all-cause mortality, CVD events, and diabetic microvascular complications, independent of high HbA1c [14, 40]. It is unclear whether the HVS is associated with the risk of aortic stiffness progression in T2D participants. In this study, restricted cubic splines were used to explore the dose-response relationship of the HVS with aortic stiffness, and the association of the adverse outcome with the HVS categories (≥ 0 to ≤ 20, >20 to ≤ 40, >40 to ≤ 60, >60 to ≤ 80, and > 80 to ≤ 100, with the ≥ 0 to ≤ 20 as reference) was investigated with logistic regression models. Second, the SD of HbA1c and FBG was included in the sensitivity analysis. The statistical software package R (version 4.1.1) was used to perform all data analyses. A 2-sided P value less than 0.05 was considered statistically significant.

## Results

### Clinical characteristics of participants

The mean (SD) follow-up period was 2.6 (1.3) years. Table 1 displays the baseline clinical and laboratory characteristics of all participants and of those with reduction/persistently low and increase/persistently high ba-PWV values. Compared with participants who reduced/persisted with low ba-PWV values during follow-up, those with increased/persisted with high ba-PWV tended to be older; have a longer diabetes duration; have higher values of SBP, FBG, HbA1c, average FBG and HbA1c during the follow-up visit; and have a higher number of measurements. Furthermore, participants who increased/persisted with high ba-PWV values had a higher prevalence of CVD and greater use of antihypertensive agents.

**Table 1:** Baseline clinical-laboratory characteristics of all participants and grouped according to serial ba-PWV measurements (reduction/persistently low or increase/persistently high) during follow-up

Characteristics	All participants (n = 2115)	Participants with reduction/persistently low ba-PWV (n = 1068)	Participants with increase/persistently high ba-PWV (n = 1047)	*P* value
Male (%)	1261 (59.62%)	653 (61.14%)	608 (58.07%)	0.163
Age (years)	56.25 (10.78)	52.70 (11.34)	59.86 (8.82)	< 0.001
Diabetes duration (months)	95.94 (88.36)	82.60 (81.57)	109.55 (92.88)	< 0.001
Ideal smoking (%)	1658 (78.39%)	822 (76.97%)	836 (79.85%)	0.120
Alcohol consumption (%)	213 (10.07%)	103 (9.64%)	110 (10.51%)	0.558
History of CVD (%)	277 (13.10%)	113 (10.58%)	161 (15.38%)	0.001
BMI (kg/m^2^)	25.75 (3.60)	25.88 (3.74)	25.61 (3.45)	0.083
DBP (mmHg)	74.72 (10.76)	74.85 (10.98)	74.59 (10.54)	0.584
SBP (mmHg)	129.56 (17.59)	127.24 (17.03)	131.92 (17.85)	< 0.001
Heart rate (bpm)	85.43 (12.58)	85.88 (12.56)	84.97 (12.59)	0.096
Baseline FBG (mmol/L)	8.89 (2.91)	8.76 (2.88)	9.02 (2.93)	0.041
Average FBG (mmol/L)	7.89 (1.45)	7.78 (1.45)	8.01 (1.45)	< 0.001
Baseline HbA1c (%)	7.74 (1.65)	7.64 (1.63)	7.83 (1.67)	0.008
Average HbA1c (%)	7.01 (0.80)	6.93 (0.88)	7.09 (0.87)	< 0.001
UA (µmol/L)	328.28 (79.74)	329.18 (78.60)	327.61 (81.13)	0.652
TG (mmol/L)	1.91 (1.69)	1.97 (1.78)	1.85 (1.60)	0.101
TC (mmol/L)	4.96 (1.17)	4.99 (1.13)	4.93 (1.20)	0.215
HDL (mmol/L)	1.26 (0.31)	1.25 (0.31)	1.27 (0.32)	0.090
LDL (mmol/L)	3.05 (0.95)	3.06 (0.93)	3.03 (0.98)	0.368
UACR (mg/mmol)	7.73 (29.82)	7.39 (31.99)	8.09 (28.12)	0.591
1st ba-PWV measurement (cm/s)	1636.70 (355.06)	1527.66 (304.00)	1747.72 (368.79)	< 0.001
Last ba-PWV measurement (cm/s)	1704.04 (370.33)	1459.26 (193.61)	1953.27 (339.86)	< 0.001
Number of HbA1c measurements	8.23 (4.10)	7.72 (3.91)	8.76 (4.23)	< 0.001
Number of FBG measurements	8.18 (4.09)	7.67 (3.91)	8.69 (4.21)	< 0.001
Insulin, n (%)	462 (21.84%)	215 (20.13%)	247 (23.59%)	0.061
Oral antidiabetic agents, n (%)	1623 (76.74%)	803 (75.19%)	820 (78.32%)	0.098
lipid-lowering agents, n (%)	539 (25.48%)	255 (23.88%)	284 (27.13%)	0.096
Antihypertensive agents, n (%)	1017 (48.09%)	434 (40.64%)	583 (55.68%)	< 0.001

Results are expressed as numbers (percentages) for categorical variables or as mean (SD) for continuous variables. DBP Diastolic blood pressure, SBP Systolic blood pressure, BMI Body mass index, CVD Cardiovascular disease, FBG Fasting blood glucose, HbA1c Glycated hemoglobin, UA Uric acid, TG Triglycerides, TC Total cholesterol, HDL High-density lipoprotein, LDL Low-density lipoprotein, UACR Urinary albumin-to-creatinine ratio, ba-PWV brachial-ankle pulse wave velocity

### Impact of HbA1c and FBG trajectories on aortic stiffness progression

We identified four distinct trajectories of HbA1c changes across the follow-up period: Low (1455, 68.79%), Median (98, 4.63%), U-shape (132, 6.24%), Decreasing (430, 20.33%) (Fig. 1). Likewise, four trajectories of FBG were identified, and labeled as Low (1387, 65.58%), Median (418, 19.76%), U-shape (228, 10.78%), and Inverse U-shape (82, 3.88%) (Fig. 1). Table 2 presents the ORs and 95% CIs of HbA1c and FBG trajectories for aortic stiffness progression in T2D participants. For HbA1c, compared with the reference (Low) class, the unadjusted ORs (95% CI) were 1.03 (0.64, 1.38), 2.07 (1.53, 2.77), and 1.02 (0.78, 1.19) for the Median, U-shape and Decreasing trajectories, respectively. After adjusting for baseline covariates including age, gender, diabetes duration, SBP, heart rate, BMI, ideal smoking, alcohol consumption, history of CVD, LDL, TG, UACR, and the use of antihypertensive agents, lipid-lowering agents, insulin, or oral antidiabetic agents, and average HbA1c during follow-up, the U-shaped trajectory had 2.17-fold (95% CI, 1.65–2.83) odds of aortic stiffness compared with the Low trajectory. Similarly, the U-shaped trajectory of FBG remained significantly associated with the outcome after adjusting for potential confounders, with an OR of 1.21 (95% CI, 1.04–1.65) (Table 2).

**Table 2:** OR and 95% CI of HbA1c and FBG Trajectory Classes on aortic stiffness progression in the T2D participants

	Model 1		Model 2		Model 3	
	OR (95% CI)	*P*	OR (95% CI)	*P*	OR (95% CI)	*P*
HbA1c Trajectory						
Low	ref.		ref.		ref.	
Median	1.03 (0.64–1.38)	0.582	1.04 (0.86–1.37)	0.504	0.98 (0.68–1.27)	0.683
U-shape	2.07 (1.53–2.77)	< 0.001	2.21 (1.79–2.94)	< 0.001	2.17 (1.65–2.83)	< 0.001
Decreasing	1.02 (0.78–1.19)	0.593	1.07 (0.85–1.19)	0.217	1.01 (0.78–1.32)	0.522
FBG Trajectory						
Low	ref.		ref.		ref.	
Median	1.11 (0.80–1.54)	0.530	1.03 (0.87–1.21)	0.364	1.02 (0.75–1.19)	0.421
U-shape	1.33 (1.09–1.62)	0.005	1.24 (1.01–1.53)	0.019	1.21 (1.04–1.65)	0.022
Inverse U-shape	1.06 (0.68–1.39)	0.433	1.09 (0.81–1.45)	0.253	1.05 (0.73–1.47)	0.376

Model 1: Adjusting for age, gender

Model 2: Adjusting for variables in model 1 plus diabetes duration, SBP, heart rate, BMI, ideal smoking, alcohol consumption, history of CVD, LDL, TG, UACR, and use of antihypertensive agents, lipid-lowering agents, insulin, or oral antidiabetic agents

Model 3: Adjusted for variables in model 2 plus average HbA1c during follow-up

### Associations of visit-to-visit variability in HbA1c and FBG with aortic stiffness progression

**Fig. 2:**
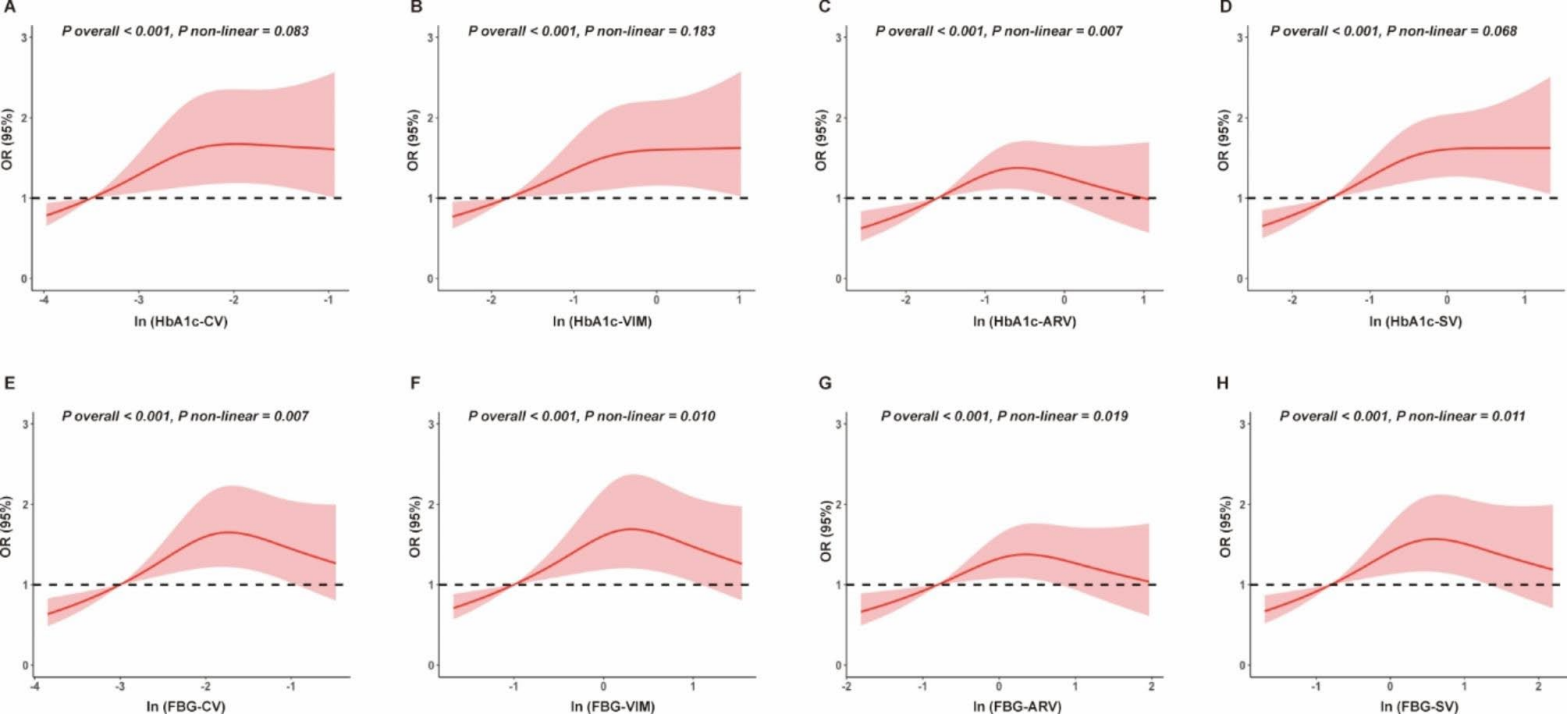
Association of predicted glycemic variability with aortic stiffness progression. A-D. The odds of aortic stiffness progression in relation to HbA1c CV, HbA1c SV, HbA1c VIM, and HbA1c ARV. E-H. The odds of aortic stiffness progression in relation to FBG CV, FBG SV, FBG VIM, and FBG ARV. The solid lines indicate multivariate-adjusted ORs and the corresponding red background indicates the 95% CI derived from restricted cubic spline regression. A knot is located at the 10th, 50th, and 90th percentiles for HbA1c or FBG variability

The role of the U-shaped trajectories of HbA1c and FBG allowed us to further investigate the impact of glycemic variability on aortic stiffness progression. Regarding HbA1c, the odds of aortic stiffness progression significantly increased with increases in the CV, SV, and VIM linearly (P nonlinear > 0.05; Fig. 2A, B, D), while a nonlinear dose-response pattern (P nonlinear = 0.007) was observed between ARV and aortic stiffness progression (Fig. 2C). Regarding FBG, four variability indices showed nonlinear dose-response relationships with aortic stiffness progression (P overall < 0.001, P nonlinear < 0.05; Fig. 2E-F). After natural log-transformations of the indices of HbA1c and FBG variability, the HbA1c variability indices were significantly associated with the progression in aortic stiffness (P < 0.05) in multivariate-adjusted logistic regression models (Table 3). Even after further adjustment for average HbA1c during follow-up visits, HbA1c variability indices, except for ARV, remained significantly (P < 0.05) associated with arterial stiffness progression, with ORs ranging from 1.20 to 1.24. In contrast, the significant association between FBG variability and aortic stiffness was diminished after adjusting for average HbA1c during follow-up visits (Table 3). A cross-tabulation analysis of HbA1c mean and VIM tertiles concerning aortic stiffness was conducted (Table 4). Patients with the third tertile of the HbA1c VIM and the third tertile of the average HbA1c had the highest odds of aortic stiffness (OR: 1.78, 95% CI: 1.23–2.58; p = 0.010).

**Table 3:** OR and 95% CI of HbA1c and FBG variability on aortic stiffness progression in the T2D participants

	Model 1		Model 2		Model 3	
	OR (95% CI)	*P*	OR (95% CI)	*P*	OR (95% CI)	*P*
HbA1c Variability (%)						
ln (HbA1c-CV)	1.33 (1.14–1.56)	< 0.001	1.32 (1.12–1.56)	< 0.001	1.24 (1.04–1.49)	0.016
ln (HbA1c-VIM)	1.27 (1.09–1.47)	0.002	1.27 (1.09–1.48)	0.003	1.22 (1.05–1.43)	0.011
ln (HbA1c-ARV)	1.26 (1.10–1.44)	< 0.001	1.25 (1.08–1.44)	0.002	1.16 (0.98–1.38)	0.084
ln (HbA1c-SV)	1.28 (1.12–1.45)	< 0.001	1.27 (1.11–1.46)	< 0.001	1.20 (1.03–1.41)	0.021
FBG Variability (mmol/L)						
ln (FBG-CV)	1.28 (1.11–1.48)	< 0.001	1.27 (1.08–1.48)	0.004	1.19 (1.00-1.41)	0.051
ln (FBG-VIM)	1.22 (1.04–1.42)	0.015	1.20 (1.01–1.42)	0.036	1.16 (0.97–1.37)	0.096
ln (FBG-ARV)	1.24 (1.09–1.42)	0.001	1.22 (1.06–1.41)	0.007	1.13 (0.96–1.34)	0.150
ln (FBG-SV)	1.25 (1.10–1.42)	< 0.001	1.23 (1.07–1.42)	0.004	1.15 (0.98–1.35)	0.092

Model 1: Adjusting for age and gender

Model 2: Adjusting for variables in model 1 plus diabetes duration, SBP, heart rate, BMI, ideal smoking, alcohol consumption, history of CVD, LDL, TG, UACR, and use of antihypertensive agents, lipid-lowering agents, insulin, or oral antidiabetic agents

Model 3: Adjusted for variables in model 2 plus average HbA1c during follow-up

**Table 4:** Cross-tabulation of HbA1c mean and VIM tertiles in relation to aortic stiffness progression

Tertiles of ln(HbA1c-VIM)	T1 of mean (≤ 6.58%)	T2 of mean (6.59–7.23%)	T3 of mean(> 7.23%)
**T1 of ln(HbA1c-VIM) (≤ -0.91)**	Ref.	1.13 (0.74–1.73)	1.45 (1.03–2.16)
**T2 of ln(HbA1c-VIM) (-0.91 to -0.42)**	1.15 (0.79–1.66)	1.51 (1.04–2.33)	1.60 (1.08–2.37)
**T3 of ln(HbA1c-VIM) (> -0.42)**	1.32 (0.87–2.01)	1.47 (1.00-2.17)	**1.78 (1.23–2.58)**

Data are displayed as OR and 95% CI. T1, tertile 1; T2, tertile 2; T3, tertile 3. Adjusting for age, gender, diabetes duration, SBP, heartrate, BMI, ideal smoking, alcohol consumption, history of CVD, LDL, TG, UACR, and use of antihypertensive agents, lipid-lowering agents, insulin, or oral antidiabetic agents

### Sensitivity analysis

Sensitivity analysis was conducted using the HVS and SD of HbA1c and FBG over the follow-up period. Restricted cubic spline regression showed that the odds of aortic stiffness progression increased linearly with elevated HVS and HbA1c SD (P nonlinear > 0.05), and nonlinearly with FBG SD (P nonlinear = 0.010) (Supplemental Fig. 3). Furthermore, HbA1c SD, but not FBG SD, showed a significant association with aortic stiffness progression according to the multivariate logistic regression models (Supplemental Table 1). Compared with the reference, the highest HVS group (> 80 to ≤ 100) was positively associated with the odds of aortic stiffness progression, independent of the average HbA1c (OR: 1.71, 95% CI: 1.25–2.64; p < 0.001).

## Discussion

This prospective cohort study demonstrated that HbA1c variability, but not FBG variability, was significantly associated with increased odds of aortic stiffness progression, independent of other possible confounding factors and even the average HbA1c during the whole follow-up period; thus, HbA1c variability may contribute unique valuable for predicting aortic stiffness.

To the best of our knowledge, this is the first study describing the association of HbA1c and FBG trajectories with aortic stiffness progression assessed by ba-PWV changes. Previous studies exploring the association between glucose levels and aortic stiffness have been mainly based on a single measure of FBG or HbA1c, neglecting the potential effect of blood glucose fluctuations over time [41–43]. Failure to consider the cumulative average and fluctuating glucose levels over time may bias the true relation between glycemia and aortic stiffness risk toward the null hypothesis [44]. Thus, prospective studies evaluating the effects of long-term HbA1c and FBG trajectories on aortic stiffness progression are needed. A recent study found that greater long-term variability in FBG was associated with an increased risk of arterial stiffness in Chinese adults [45], which is partly consistent with our findings. However, the study focused on the impact of FBG variability on general adults and did not evaluate the effect of long-term variability in HbA1c on arterial stiffness in the T2D populations. Furthermore, their study did not include baseline ba-PWV measurements, instead using the second and third ba-PWV datapoints obtained during the follow-up period to define the relative change in ba-PWV as the outcome. In contrast, we obtained and categorized the baseline and final ba-PWV measurements into quartiles, and then classified the aortic stiffness progression or regression as increased/persistently high or reduced/persistently low, which better elucidated the process of arterial stiffness in T2D.

In our work, compared with those in the Low, Median, or Decreasing HbA1c trajectories, individuals with U-shaped trajectories of HbA1c and FBG had a 2.17- and 1.21-fold odds of increased aortic stiffness during the mean follow-up period of 2.6 years, respectively. The results indicated that glucose swings may play a greater role in the development of aortic stiffness. Thus, in terms of diabetes management, the use of HbA1c as the gold standard for long-term glycemic control may sometimes be misleading because HbA1c data do not provide information on hyperglycemia and hypoglycemia for participants with high glycemic variability [46]. In the current study, we log-transformed the glycemic variability indices to conform them to linear models [47], and found that HbA1c variability metrics (CV, SV, and VIM) were positively associated with aortic stiffness progression. Interestingly, the statistically significant result for U-shaped FBG (Table 2) appears to be unreconciled with the null result for FBG variability in Table 3. The possible explanation was that the methods, the population, the number of participants, and the reference during the data analysis in the two results were different. Moreover, the cross-tabulation analysis further demonstrated that individuals with higher HbA1c levels and greater variability had the highest odds of arterial stiffness progression, indicating that aortic stiffness regression might be facilitated by maintaining the target glycemic level and stable glycemic management.

However, compared with HbA1c variability metrics, FBG variability metrics were not significantly associated with adverse outcomes. These results suggested that HbA1c variability performs better than FBG variability in the prediction of aortic stiffness progression, possibly because FBG reflects immediate blood glucose levels and thus is more susceptible to day-to-day changes in other factors, such as diet, exercise, moods, and medications. Moreover, FBG does not capture postprandial glucose excursions, which are a major driver of glycemic variability; in contrast, HbA1c reflects the average blood glucose level in three months. Notably, in prior studies, FBG variability was also linked to aortic stiffness and even CVD event [45, 48]. A prospective cohort analysis including 4,982 participants in the Antihypertensive and Lipid-Lowering Treatment to Prevent Heart Attack Trial (ALLHAT) found greater variability of FBG (SD and VIM) are associated with increased mortality risk [10]. Thus, the potential roles of HbA1c and FBG variability in aortic stiffness in T2D participants should be further examined in future studies. Notably, epidemiologic studies provide evidence that CVD events were influenced by variability in other cardiovascular risk factors, such as BP [49]. Higher BP variability increased the risk of heart failure in individuals with T2D, and showed a worse prognosis in patients with myocardial infarction in a U-shaped manner, independent of the mean BP [50, 51]. These findings revealed that optimizing metabolic factors treatment strategies was warranted in the future for the reduction of cardiovascular outcomes.

These findings indicated that stable glycemic control may benefit the attenuation in aortic stiffness, and HbA1c variability performed better in the magnitude of the association with aortic stiffness progression than FBG variability. In addition, the study also raise the possibility that HbA1c variability is a useful index for identifying future risks of progression of aortic stiffness in participants with T2D. Nevertheless, future prospective investigations are needed to determine whether reducing HbA1c variability is associated with a better prognosis for aortic de-stiffening (reduced aortic stiffness) in participants with T2D.

The underlying pathological mechanisms linking glycemic fluctuations and aortic stiffness progression have not been understood fully. Hyperglycemia results in vascular damage, possibly mediated by the formation of excessive advanced glycation end products and the activation of oxidative stress [52]. Antonio et al. demonstrated that oscillations in glucose levels had a more specific triggering effect than constant high glucose levels on plasma 3-nitrotyrosine and 24-h urinary excretion rates of free 8-iso prostaglandin F2 (PGF2), two well-recognized markers of oxidative stress [53]. Another study found that endothelial cells exposed to intermittent high glucose-stimulated greater production of reactive oxygen species overproduction with protein kinase C (PKC)-dependent activation of the NAD(P)H oxidase pathway, leading to the development of vascular injury in diabetes [54]. A plausible explanation for those findings is that chronic exposure to high, stable glucose levels provides sufficient time to trigger metabolic compensation mechanisms, whereas this adaptation might be lacking or significantly reduced in response to intermittent high glucose [54]. Additionally, Risso et al. designed an experimental study: human umbilical vein endothelial cells were incubated in media with different glucose concentrations media for 14 days; they found that apoptosis-related protein levels were significantly enhanced in these cells when exposed to intermittent, rather than constant, high glucose concentrations, which demonstrates that apoptosis may be another mechanism mediating the deleterious effect of glycemic variability on endothelial cells [55].

Notably, in addition to acute hyperglycemia, hypoglycemia might also be an independent cause of diabetic vascular complications. As we described above, in the ACCORD trial, the use of aggressive glycemic treatment to target normal HbA1c levels elevated the risk of mortality, and did not significantly reduce major CVD events in participants with T2D [9]. Similarly, the Degludec vs. Insulin Glargine in Participants with T2D at High Risk of Cardiovascular Events Trial recently suggested that higher day-to-day fasting glycemic variability is significantly associated with increased risks of severe hypoglycemia and all-cause mortality [56]. Inflammatory cytokines overproduction, activation of the sympato-adrenal response, endothelial dysfunction, and blood coagulation abnormalities are possible potential mechanisms by which hypoglycemia could elevate the risk of vascular disease in diabetes [57–59].

The strengths of our study were clear. First, the complete follow-up data provided a relatively large number of HbA1c and FBG measurements that allowed us to accurately calculate glycemic variability with several variability metrics. Second, our results compared the impact of variability in two different glycemic indices (HbA1c and FBG) on aortic stiffness and demonstrated that HbA1c shows better performance for predicting the odds of aortic stiffness progression than FBG in participants with T2D regardless of the average glycemic level during the follow-up period. However, several limitations in the present study should be noted. First, the aortic stiffness progression was defined as the changes in ba-PWV values during the whole follow-up period; however, the role of PWV measured by carotid-femoral PWV should be considered, because it could also be used to assess regression or progression in aortic stiffness [33, 34]. Therefore, the results might not be generalizable to PWV in other regions. Second, the relatively short follow-up period might lead to a small bias in interpreting our results, although there were sufficient HbA1c and FBG measurements to calculate glycemic variability (mean number of measurements: 8.2) during the follow-up visits. A longer follow-up period would be better for elucidating the correlations between glycemic variability and aortic stiffness in the future.

## Conclusions

In summary, this prospective study provides new evidence that HbA1c variability is significantly associated with the odds of aortic stiffness progression evaluated by ba-PWV values in individuals with T2D, independent of the average HbA1c. Moreover, the use of the HbA1c trajectory enables feasible and early identification of individuals who are at high odds of aortic stiffness progression, reducing or preventing the development of subclinical atherosclerosis. In clinical practice, stable glycemic control is recommended for T2D participants. Nevertheless, the mechanisms underlying the relationship between glycemic variability and subclinical atherosclerosis need to be elucidated in future studies.

## Electronic supplementary material

Below is the link to the electronic supplementary material.

Supplementary Material 1

Supplementary Material 2

Supplementary Material 3

Supplementary Material 4

## Data Availability

The datasets generated and/or analyzed during the current study are not publicly available because of the individual privacy of the participants but are available from the corresponding author on reasonable request.

## Abbreviations

ABI: Ankle-brachial pressure index
ACCORD: Action to Control Cardiovascular Risk in Diabetes
ARV: Average real variability
Ba-PWV: Brachial-ankle pulse wave velocity
BMI: Body mass index
BP: Blood pressure
CGM: Continuous glucose monitoring
CI: Confidence interval
CVD: Cardiovascular disease
CV: Coefficient of variation
FBG: Fasting blood glucose
GPO–POD: Glycerophosphate oxidase–peroxidase
HbA1c: Glycated hemoglobin
HVS: HbA1c variability score
LDL: Low-density lipoprotein cholesterol
MMC: Metabolic Management Center
MICEs: Multiple imputation of chained equations
PGF2: Prostaglandin F2
OR: Odds ratio
PKC: Protein kinase C
SBP: Systolic blood pressure
SD: Standard deviation
SV: Successive variation
TG: Triacylglycerols
T2D: Type 2 diabetes
UACR: Urinary albumin/creatinine ratio
VIM: Variability independent of the mean
WHO: World Health Organization.
