# Supplementary figures and images for: Visit-to-visit HbA1c variability is associated with aortic stiffness progression in participants with type 2 diabetes
Source: Cardiovasc Diabetol. 2023 Jul 6;22:167. doi: 10.1186/s12933-023-01884-7 (PMC10324236; doi:10.1186/s12933-023-01884-7)

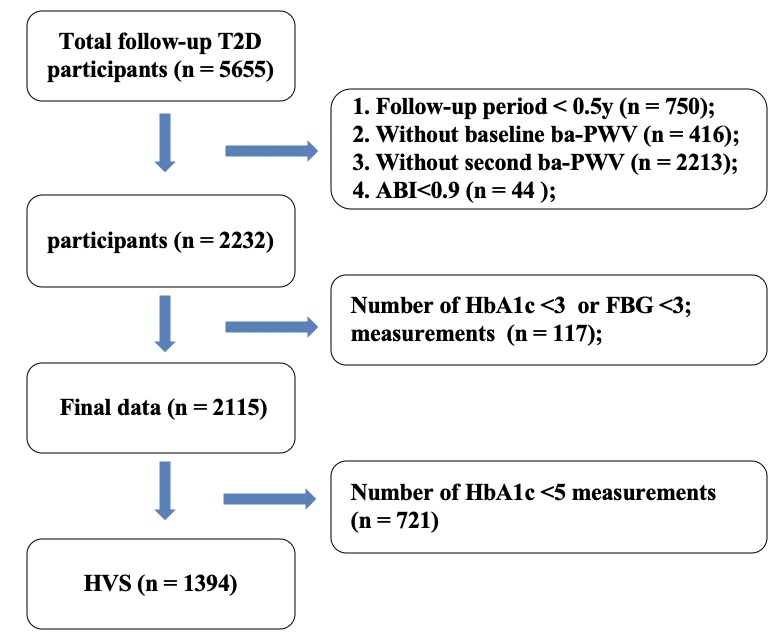

Supplement: Supplementary file 1 — Supplementary Material 1 [file 12933_2023_1884_MOESM1_ESM.png]

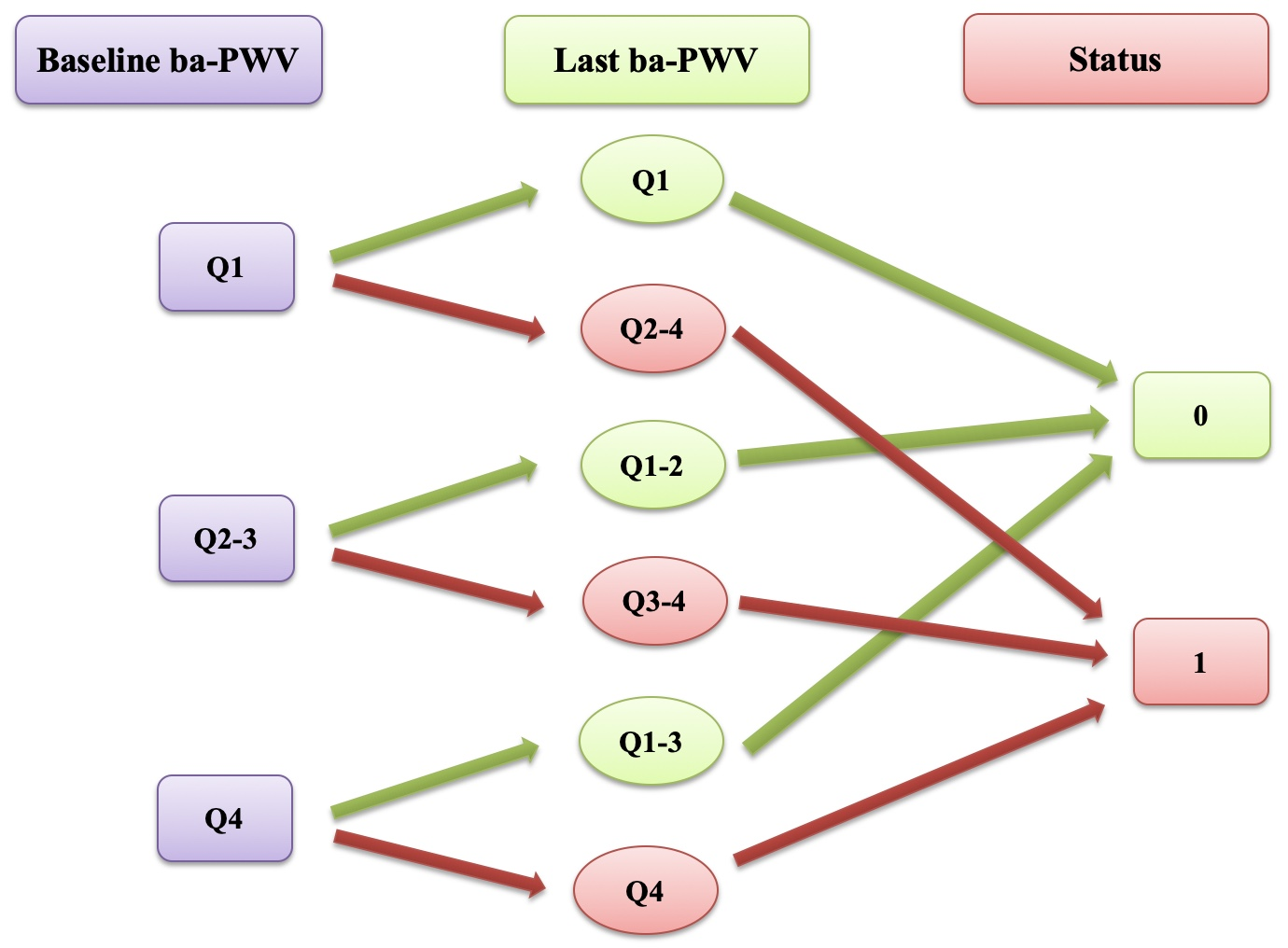

Supplement: Supplementary file 2 — Supplementary Material 2 [file 12933_2023_1884_MOESM2_ESM.png]

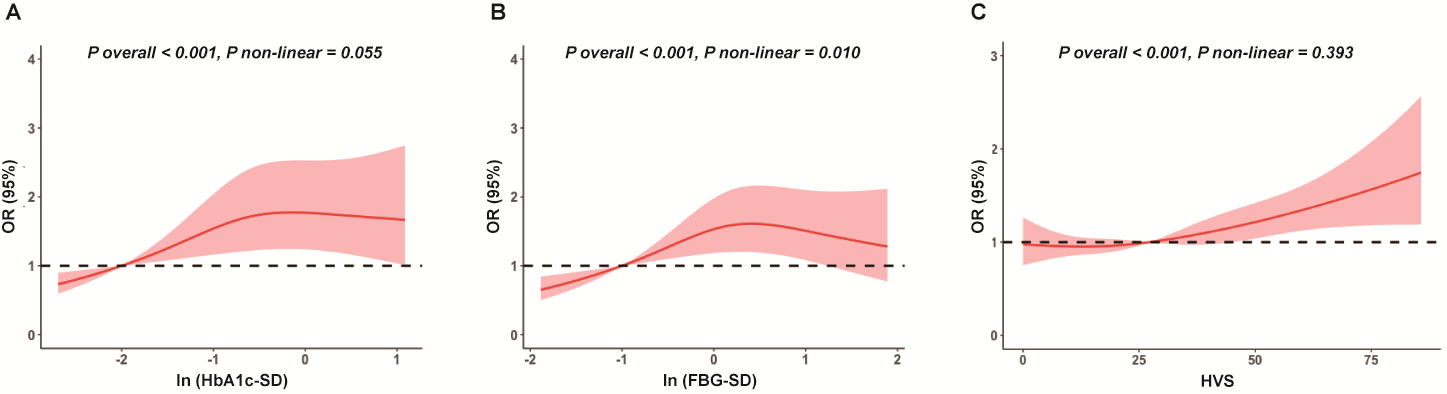

Supplement: Supplementary file 3 — Supplementary Material 3 [file 12933_2023_1884_MOESM3_ESM.png]
