# Supplementary material for: Visit-to-visit HbA1c variability is associated with aortic stiffness progression in participants with type 2 diabetes
Source: Cardiovasc Diabetol. 2023 Jul 6;22:167. doi: 10.1186/s12933-023-01884-7 (PMC10324236; doi:10.1186/s12933-023-01884-7)
Supplement: Supplementary file 4 — Supplementary Material 4 [file 12933_2023_1884_MOESM4_ESM.docx]

Supplemental Table 1. OR and 95% CI of HVS categories on aortic stiffness progression in the T2D participants.

|  |  | **Model 1** | | **Model 2** | | **Model 3** |  |
| --- | --- | --- | --- | --- | --- | --- | --- |
|  | n | **OR (95% CI)** | **P value** | **OR (95% CI)** | **P value** | **OR (95% CI)** | **P value** |
| **ln(HbA1c-SD)** | 2115 | 1.29 (1.14-1.47) | < 0.001 | 1.29 (1.13-1.48) | < 0.001 | 1.23 (1.05-1.44) | 0.010 |
| **ln(FBG-SD)** | 2115 | 1.27 (1.12-1.44) | < 0.001 | 1.26 (1.10-1.45) | 0.001 | 1.18 (0.99-1.39) | 0.054 |
| **HVS Categories** |  |  |  |  |  |  |  |
| HVS (0-20) | 603 | Ref. |  | Ref. |  | Ref. |  |
| HVS (20-40) | 396 | 1.53 (1.17-2.00) | 0.002 | 1.49  (1.12-1.98) | 0.005 | 1.36 (1.01-1.82) | 0.042 |
| HVS (40-60) | 275 | 1.65 (1.22-2.24) | 0.001 | 1.62 (1.18-2.22) | 0.003 | 1.36 (1.03-1.86) | 0.035 |
| HVS (60-80) | 100 | 1.67 (1.31-2.33) | < 0.001 | 1.64 (1.21-2.23) | < 0.001 | 1.52 (1.13-2.03) | 0.002 |
| HVS (> 80) | 20 | 2.16 (1.34-4.13) | < 0.001 | 2.35 (1.43-4.25) | < 0.001 | 1.71 (1.25-2.64) | < 0.001 |
| **P for trend** | 1394 |  | < 0.001 |  | < 0.001 |  | < 0.001 |

Model 1: Adjusting for age and gender.

Model 2: Adjusting for variables in model 1 plus diabetes duration, SBP, heart rate, BMI, ideal smoking, alcohol consumption, history of CVD, LDL, TG, UACR, and use of antihypertensive agents, lipid-lowering agents, insulin, or oral antidiabetic agents.

Model 3: Adjusted for variables in model 2 plus average HbA1c during follow-up.

Supplemental formula:

$1. HbA1c-VIM=\frac{k * SD (HbA1c)}{Mean{(HbA1c)}^{x}}$ $k =Mean({Mean(HbA1c)}^{x}$

$2. HbA1c-CV=\frac{SD (HbA1c)}{Mean(HbA1c)}*100\%$

$3. HbA1c-ARV=(\frac{1}{n-1})\sum_{i=1}^{n-1} |{HbA1c}_{i+1}-{HbA1c}_{i}|$

*4.* $HbA1c-SV=\sqrt{(1/(n-1))\sum_{(i=1)}^{(n-1)} {({BP}_{i+1}-{BP}_{i})}^{2}})$

$$5. HVS=\frac{Number of HbA1c fluctuation events (\Delta>0.5\%)}{Total number of HbA1c measurements-1}x100$$
